# Skin tissue engineering: wound healing based on stem-cell-based therapeutic strategies

**DOI:** 10.1186/s13287-019-1212-2

**Published:** 2019-03-29

**Authors:** Azar Nourian Dehkordi, Fatemeh Mirahmadi Babaheydari, Mohammad Chehelgerdi, Shiva Raeisi Dehkordi

**Affiliations:** 1 Department of Stem Cell and Regenerative Medicine, Institute of Medical Biotechnology, National Institute of Genetic Engineering and Biotechnology, Tehran, Iran; 2 Cellular and Molecular Research Center, Basic Health Sciences Institute, Shahrekord University of Medical Sciences, Shahrekord, Iran; 3 Biotechnology Research Center, Shahrekord Branch, Islamic Azad University, Shahrekord, Iran; 4 Department of Genetics, Faculty of Science, Shahrekord University, Shahrekord, Iran

**Keywords:** Wound healing, Cytokines, Chronic wounds, Growth factors, Stem cells, Skin

## Abstract

Normal wound healing is a dynamic and complex multiple phase process involving coordinated interactions between growth factors, cytokines, chemokines, and various cells. Any failure in these phases may lead wounds to become chronic and have abnormal scar formation. Chronic wounds affect patients’ quality of life, since they require repetitive treatments and incur considerable medical costs. Thus, much effort has been focused on developing novel therapeutic approaches for wound treatment. Stem-cell-based therapeutic strategies have been proposed to treat these wounds. They have shown considerable potential for improving the rate and quality of wound healing and regenerating the skin. However, there are many challenges for using stem cells in skin regeneration. In this review, we present some sets of the data published on using embryonic stem cells, induced pluripotent stem cells, and adult stem cells in healing wounds. Additionally, we will discuss the different angles whereby these cells can contribute to their unique features and show the current drawbacks.

## Background

Skin ulcers develop in the case of tissue disintegration and are caused by many different factors, from long-term pressure or lack of circulation and trauma [1]. The process healing of skin ulcers is composed of the coordination of three overlapping but distinct phases. This includes inflammation, proliferation and remodeling. The wound-healing process is highly regulated by secretion of various growth factors, cytokines, and chemokines [2]. Disruption of the cellular and molecular signals of these stages can lead to chronic wound formation [3]. Common wound care involves selecting appropriate dressings to maintain a favorable wound-healing environment, control infection control, debride the tissue, and address the underlying causes such as ischemia and diabetes. Nevertheless, the efficacy of the current treatment modalities is a limited success and incurs considerable costs. The approach of regenerative medicine has emerged as an alternative to provide additional therapeutic options to potentially improve wound healing and restore normal skin architecture [4–6]. Stem cell-based therapy has become a promising new approach in the field of regenerative medicine. The considerable interest in the biology of stem cells is concerned with their capacity for self-renew and differentiate multiple cell types and is crucial for physiologic tissue renewal and regeneration after injury [7]. Researchers would anticipate achieving acceleration in healing, earlier wound closure, prevention of wound contracture and scar formation, and ideally regeneration of the skin using stem cell administration. However, determining the optimum source, a method of processing and administration from the clinical standpoint, as well as defining the roles of stem cells in the real clinical situation, is still the remaining challenge for using stem cells for their regenerative wound healing [8]. In this review, we will discuss the use of stem cells in skin regeneration.

## Anatomy of skin

The skin is considered one of the most vital organs in the body due to its important functions as a protective barrier against various external agents and a temperature regulator [9]. It is made up of three layers: epidermis, dermis, and hypodermis (subcutaneous layer**)** [10]. The epidermis forming the outermost layer provides a waterproof barrier playing a crucial role in controlling the moisture into the body [11]. Keratinocytes are the most abundant cells present in the epidermis (approximately 90%), whereas the remaining population of epidermal cells was occupied by melanocytes, Langerhans cells, and Merkel cells [12]. The epidermis is generally composed of several layers according to keratinocytes morphology. In fact, keratinocytes are formed by division germ cells (basal cells) at the basal cell layer while migrating through a granular layer to the upper epidermal layers to form a dead cell on the surface of the skin [13]. Dermis accounts for approximately 90% of the weight of the skin and forms the foundation of this organ system [14]. The dermis represents the inner layer of the skin between the epidermis and the hypodermis [15]. The dermis is a connective tissue consisted of extracellular matrix (ECM), vascular endothelial cells, and fibroblasts, along with adipose glands, sweat glands, hair follicles, blood vessels, and nerve endings. Fibroblasts are the main dermal cell population releasing collagen and elastin, resulting in mechanical strength and elasticity of the skin [16]. The hypodermis is the deepest layer of the skin consisting of loose connective tissue, and cells storing fat (half of the body fats stored in this place), blood vessels, and nerves. The tissue is especially rich in proteoglycan and glycosaminoglycans absorbing fluid into the tissue (holds the water inside) and giving it mucous-like properties [17].

## Wounds

The wound is described as injury and any disorder in the normal structure of the skin, which can cause loss of conjunction in the body tissue [18]. One of the most substantial proceedings in healing each wound should identify the cause of the wound. As a result, the wounds are defined as open and closed wounds based on their nature [19]. Time is a significant parameter in wound healing and repair. Accordingly, wounds are clinically divided into two acute and chronic groups [19]. Wounds destroying the integrity of soft tissue and closure spontaneously by following timely and orderly progression (between 4 and 6 weeks) are assorted as acute wounds [20]. In addition, acute wounds can be caused by various mechanisms or environmental exposure such as extreme temperature changes, contact with chemicals, and radiation, resulting in the entrance of the organism into the body and infection. They can be graded according to their size, depth, and location [21]. Chronic wounds can be created for many reasons, including infection, physical agents, inflammation, and tumors. Unlike acute wounds, healing of chronic wounds is delayed (more than 12 weeks) due to prolonged pathological inflammation, while the process of treatment is similar in both types [22].

## The processes and characteristics of dermal wound repair

Wound repair is a normal and complex biological process in the human body occurring in all tissues and organs [23]. It depends on the type of injury, the underlying disease, systemic mediators, and local wound factors [24]. Dermal wound repair is a highly dynamic process involving interaction between epidermal and dermal cells, controlled angiogenesis, the extracellular matrix, and plasma-derived proteins (coordinated by cytokines and growth factors) [25]. The many biological mechanisms overlapping during the progression of the skin wound repair reaction can describe the loss of consensus on the number of phases involved in this reaction. However, all researchers maintain that these phases are interrelated, and suggest that the wound repair process is a continuum [26]. The immediate aim of repair is to achieve tissue homeostasis and integrity in order to accomplish this aim as the repair process consisting of three phases: inflammation, proliferation, and tissue remodeling [27]. These phases and their physiological functions occur in a regulated and precise manner since discontinuities, aberrancies, or lengthening in the process can lead to delayed wound repair or a non-repair chronic wound [28].

### Inflammation

Inflammation occurs immediately after tissue damage, and the key aim of this phase is to prevent infection [20]. Since the mechanical barrier as the frontline against exceeding microorganisms is no longer intact. The inflammatory phase is separated vascular response (hemostasis) and cellular response (inflammation) [29]. The vascular response consists of platelet activation leading to the formation of a fibrin clot and repair of the vascular system of the injured tissue. The fibrin clot is made of platelets, collagen, fibronectin, and thrombin. The fibrin clot provides a scaffold for using monocytes, neutrophils, endothelial cells, and fibroblasts [30]. The inflammatory response begins with the release of cytokines such as transforming growth factor (TGF-β), platelet-derived growth factor (PDGF), epidermal growth factor (EGF), fibroblast growth factor (FGF), and interleukin 8 (IL8/CXCL-8) from the fibrin clot and directly from the damaged tissue [29]. These work as strong chemotactic signals to recruit neutrophils to the wound. Neutrophils have various mechanisms for removing bacteria, foreign particles, and damaged tissue. They have the phagocytotic activity for ingesting and destroying foreign particles. They can also degranulate and release various toxic substances (i.e., eicosanoids, cationic peptides, and proteinases, i.e., elastase, proteinase 3, cathepsin G, and an urokinase-type plasminogen activator), which will remove bacteria and dead host tissue. Oxygen-derived free radical species have bactericidal properties produced as a by-product of neutrophil activity [20]. Approximately 3 days after injury, neutrophils decrease in number and are replaced with macrophages. They have many tasks such as promotion and resolution of inflammation, host defense, removal of apoptotic cells, and support of cell proliferation and tissue restoration [2].

### Proliferation

The proliferative stage of healing arises approximately 2–10 days after wounding and is determined through interaction between different cell types. Initially, keratinocytes are called to the injured dermis and then the angiogenesis occurs. During angiogenesis, capillary sprouts accompanied by fibroblasts and macrophages replace the fibrin clot, which is termed as granulation tissue. Various factors are involved in this process among which vascular endothelial growth factor (VEGF) and FGF play a focal role in the regulation [20]. Furthermore, angiogenesis is triggered by stimulation of the bone marrow and endothelial progenitors at normal concentrations of oxygen. In the final stage, fibroblasts derived around a wound or bone marrow motivated through macrophages are transformed into myofibroblasts. Myofibroblasts are identified as contractile cells and play a remarkable role in the closure of the wound [31]. Both fibroblasts and myoblasts synthesize and deposit ECM proteins, predominantly in the form of collagen that eventually forms a scar [32]. It is crucial to maintain a balance between ECM protein deposition and degradation, since the disruption of this process causes abnormalities in scarring [33].

### Remodeling

The final phase of healing consists of remodeling, which begins 2–3 weeks after injury and continue up to 2 years or more [30]. The main goal of the remodeling stage is to extend new epithelium and apoptosis of unneeded blood vessels, fibroblasts, and inflammatory cells, resulting in maturation of scar [2]. During this stage, the composition of matrix alters, and type III collagen is eradicated and replaced with type I collagen, which is performed by matrix metalloproteinase (MMP) produced by fibroblasts, macrophages, and endothelial cells to strengthen the scar [34].

## Scarless healing of fetal wounds

Fetal wound scarless healing is known as an ideal method of healing. This method is completed by some combined actions within cells such as adhesion molecules, cytokines under the precise control of genes, and the ECM [35]. ECM synthesis and remodeling are essential for wound healing; recent studies have focused on the differences between fetal and adult fibroblasts and have revealed that in collagen gels in compression with adult fibroblasts, fetal fibroblasts have a greater migratory capacity, more hyaluronate receptors, and different growth factor profiles [36]. Studies into dermal matrices containing fetal and adult fibroblasts have indicated that dermal matrices containing fetal fibroblasts could promote scarless repair. A number of upregulated anti-fibrotic genes and downregulated fibrotic genes have been detected in the fetal dermal matrix [37]. The results obtained by Hu et al. revealed that in keratinocytes and fibroblasts between scarless and scarring wound, 546 genes had differential expression. They also identified more than 60 differential pathway regulations in scarless and scarring skin cells in fetal murine [38]. Owing to high expansion capacity under simple culture conditions, engineering of fetal tissue has high potential for the treatment of human skin wounds [39]. FcgammaRIII-X protein containing 249 amino acid that is beneficial for embryonic scar-free healing has been known as a transcript in embryonic cells with a positive regulatory role in wound healing [40]. Research studies have indicated fetal wound healing have a different inflammatory response than adult wound healing [41]. The levels of immune cells, i.e., macrophages, are reduced and less activated in fetal wound healing compared to immune cells in adult wound healing. Furthermore, in fetal wound healing, the presence of inflammatory cells is short-lived compared to adult wound healing [42], indicating that the reduced number of inflammatory cells means lower expression levels of some growth factors and cytokines over a shorter time [43, 44]. There are some fetal regeneration differences compared to scar-forming healing, which may result in new therapeutic targets to prevent or at least reduce adult healing scarring [45].

## Dermal and nature substitute

Dermal substitutes play a crucial role in reducing scar formation in skin reconstruction, inhibiting excessive proliferation and improving softness and contracture as well as improving mechanical wear resistance. By placing these substitutes in the wound bed, it is proposed that the cells and ECM can grow and develop to complete the dermal regeneration [46]. The materials should be appropriate to supply the cells’ nutrition and enable growth and metabolism [47]. There are various materials for dermal substitutes divided into natural biological materials and synthetic polymer materials. Four best types of natural materials are collagen, chitosan, hyaluronic acid, and carboxymethyl chitosan. Collagen is available in the human and animal connective tissues. It has a high degree of cell adhesion, the ability to support cell migration, and good biodegradability. Chitosan is another natural polymer most widely used next to collagen in wound healing owing to its many advantages such as biocompatibility, biodegradability, hemostatic activity, and antibacterial properties [48, 49]. The particular flexibility and elasticity of chitosan can significantly reduce scar development when used for burn wound healing [50]. Chitosan can stimulate collagen synthesis and FGF due to the chitosan electrostatic function, which can enhance the wound-healing rate [51, 52]. The films, gels, or sponges of chitosan have recently been investigated for use in full-thickness burn wounds [53]. However, chitosan has rapid biodegradability that is often formed in wound healing [54]. In addition, an important water-soluble chitosan known as carboxymethyl chitosan is derivative having better water-solubility and biocompatibility than chitosan [53]. Hyaluronic acid by chemical crosslinking and surface modification can improve the mechanical properties and cell affinity of scaffolds [55]. According to various products developed in the last 30 years, dermal substitutes can be categories in natural dermal substitutes and artificial dermal substitutes, which some of them have been used for clinical treatment [56]. Natural dermal substitutes replicate the collagen three-dimensional structure and have excellent biocompatibility. In addition, their tissue composition is the closest one to autologous skin [57]. In summary, the success of using natural biodegradable cell matrices has been encouraging and continues to facilitate broader use in the future. However, numerous problems such as low mechanical strength, shrinkage/contraction, difficulty in handling, and risks of immunological rejection occurring with natural polymers have been caused [58].

## Factors related to dermal regeneration

To develop better substitutional medical approaches to improvement of injured skin tissues, regenerative medicine applications have been widely investigated. Regenerative therapies consist of different technological approaches such as gene targeting, stem cell treatment, soluble molecules, cell reprogramming, and tissue engineering [59]. In particular, a basic principle for these applications is using engineering techniques to facilitate a natural wound-healing cascade by providing proper physicochemical and biochemical factors [60]. A number of bioactive factors, including growth factors and cytokines, are involved in various tissue repair stages and are necessary to promote dermal regeneration. Cytokines are extracellular signaling proteins secreted by many cell types affecting the activity of other cells, including immune cells. They include interleukins, lymphokines, interferons, and tumor necrosis factor [61]. The study of cytokines in wound healing is challenging, since examination of isolated cytokine responses in the human body usually represents an oversimplification of the phenomena. Additionally, modifying the healing process by regulating the cytokine milieu is a considerable challenge, since cytokine responses depend on time and concentration in the wound bed [62]. Growth factors are signaling proteins that release at the wound site and are required for communication between various cells such as smooth muscle cells, fibroblasts, myofibroblast, keratinocytes, endothelial cells, and immune cells [63]. They can induce angiogenesis supplying oxygen and nutrients to cells transplanted for organ substitution to maintain their biological functions [64]. Different studies on human patients have confirmed that growth factors such as PDGF are involved in enhancing the wound-healing rate in acute wounds and even provide complete healing in chronic wounds [59]. Therefore, the development of regenerative medicine applications with the aid of exogenous growth factors could be an alternative clinical solution for skin regeneration.

### Transforming growth factor beta (TGFβ)

The TGF-β superfamily consists of 33 members. In mammals, mainly TGF-β1, β2 and β3 isoforms are found, but TGF-β1 predominates in cutaneous wound healing. They are produced by macrophages, keratinocytes, fibroblasts, and platelets [59]. These three isoforms share 60–80% homology and are encoded by different genes. All three isoforms are believed to bind and signal through the two TGF-β receptors (TβRI and TβRII). TβRI activates the SMAD intracellular signaling pathway through Phosphorylation of Smad2 and Smad3 binding to Smad4, translocates into the nucleus, and activates transcription of target genes [65]. TGF-β can also activate a number of nonSmad signaling pathways, including ras/MEK/ERK, which requires the heparan sulfate-containing proteoglycan (HSPG) syndecan 4; p38, which requires the HSPG β-glycan; and c-Jun N-terminal kinase (JNK), which requires focal adhesion kinase and TGF-β-activated kinase (TAK) [66]. Much of the current knowledge on TGF-β action in wound healing has been obtained from animal studies using incisional and/or excisional wound models [67]. Preclinical studies indicated a significant reduction in scarring and considerably improved dermal architecture after intradermal injection of avotermin (TGF-β3) in adult rats [59]. In adult mammals, high levels of TGFβ1 and TGFβ2 and low levels of TGFβ3 facilitate scar-forming healing, while in fetal mammals, high levels of TGFβ3 and low levels of TGFβ1 and TGFβ2 favor scar-free healing [67]. Other evidence support the involvement of TGFβ in regeneration, including using the potent small molecule inhibitor [67, 68] and experiments with zebrafish [69]. Overall, these experimental observations support the role of TGFβ signaling in wound healing, including both non-specific scar formation and tissue-specific regeneration [70].

### Vascular endothelial growth factor (VEGF)

The VEGF is the most important signaling growth factor in angiogenesis and vasculogenesis. VEGF is involved in wound healing and is secreted by platelets, macrophages, fibroblasts, and keratinocytes [59]. The VEGF family consists of VEGF-A, VEGF-B, VEGF-C, VEGF-D, and VEGF-E and placental growth factor. VEGF-A is one of the most potent proangiogenic molecules in the skin. It has been widely investigated as an exogenous cargo growth factor for skin tissue regeneration [60]. VEGF-A is a 45 kDa heterodimeric heparin-binding protein. Multiple isoforms of VEGF-A can be generated through alternative splicing. VEGF-A interacts with multiple receptors, including VEGF receptor-1 (VEGFR-1) and VEGF receptor-2 (VEGFR-2). These are tyrosine kinase receptors that differ in their ligand binding properties and tyrosine kinase activity. Although VEGF-A binds VEGFR1 with a higher affinity than VEGFR-2, VEGFR-2 exhibits stronger inherent tyrosine kinase activity [71]. VEGFR-2 is believed to be more important than the two receptors in terms of controlling endothelial cell function and regulating angiogenesis based on its superior ability to stimulate downstream signaling cascades. On ligand binding, VEGFR-2 dimerizes, resulting in kinase activation and autophosphorylation of tyrosine residues. Phosphorylation of these residues leads to activation of protein kinase B (inhibits apoptosis), the mitogen-activated protein kinase (MAPK) pathway (induces proliferation), Src kinase, focal adhesion kinase, and p38 MAPK (leads to cell migration) [72]. It has been demonstrated that VEGF acts as an important regulator of angiogenesis (physiological and pathological) by inducing proliferation of fibroblasts and endothelial cells as well as by promoting neovascularization, re-epithelialization, and collagen deposition [73]. Artificial three-dimensional scaffolds have been used as efficient dermal regeneration templates f to treat skin defects created by burns, trauma, and chronic diseases in regenerative medicine. Inadequate angiogenesis is often caused during application of such scaffolds. Tan et al. used collagen scaffolds with VEGF in a diabetic rat wound model and found that the treatment resulted in an enhanced healing rate, improved vascularization, and increased level of VEGF in the granulation tissue [74]. Using plasmid DNA encoding activated VEGF-165 in collagen-chitosan dermal equivalents to treat the full-thickness burns could result in a significantly higher number of newly formed and mature blood vessels, enabling fast regeneration of the dermis [75].

### Platelet-derived growth factor (PDGF)

The PDGF is an important biochemical mediator of wound healing and promotes cellular reactions throughout all phases of the wound-healing process. PDGF is known to improve dermal regeneration, promote local protein and collagen synthesis, and cause angiogenesis [76]. PDGF comprises a family of homodimeric or heterodimeric growth factors: PDGF-AA, PDGF-AB, PDGF-BB, PDGF-CC, and PDGF-DD. It is mainly secreted from the α-granules of the platelet, but it is also released by different cells such as keratinocytes, macrophages, fibroblasts, and endothelial cells [60]. There are two PDGF receptors (PDGFR), PDGFR-alpha (PDGFRA) and PDGFR-beta (PDGFRB), engaging several well-characterized signaling pathways such as Ras-MAPK, PI3K, and PLC- γ, which are known to be involved in multiple cellular and developmental responses [77]. Dermal fibroblasts are one of the major target cells of PDGF in initiation and propagation of skin tissue repair. They secrete PDGF-BB and express PDGFRB receptor. PDGF-BB stimulates Wnt2 and Wnt4 mRNA expression. In terms of its relevance to wound healing and skin tissue regeneration, PDGF-BB exhibits chemotactic, mitogenic, angiogenic, and stimulatory effects, leading to modification of the extracellular matrix by stimulating collagen, collagenase, and glycosaminoglycan synthesis [78]. PDGFRB targeted deletion studies into dermal fibroblasts have demonstrated its role in transducing wound-healing signals accounting for an 85% reduction of granulation tissue mass [79]. Therefore, wound treatment using exogenous PDGF has been studied by developing a cellular collagen-chitosan temporary matrix of a wound site for in vivo dermal regeneration. This study suggested that PDGF supplementation could have altering effects on oxidative events depending on the duration of the wound-healing process [80]. In another study, a combination of AMD3100 (which mobilizes marrow-derived progenitor cell) and PDGF-BB therapy has been synergistically shown to improve progenitor mobilization and trafficking, resulting in significantly improved diabetic wound closure and neovascularization [81].

### Fibroblast growth factor (FGF)

The FGFs include a family of polypeptides growth factors which have been demonstrated to have considerable capability in tissue repair and regeneration. It was originally identified to induce proliferation and differentiation in various types of the cell [82]. The interaction of FGFs with their receptor tyrosine kinases (FGFRs) in the presence of heparin/heparan sulfate (HS) proteoglycans (HSPG) as a cofactor results in activation of FGFRs by phosphorylation of tyrosine residues [83]. Activated FGFRs lead to triggering a number of signaling pathways such as the RAS/MAP kinase pathway, PI3 kinase/AKT pathway, and PLCγ pathway, resulting in specific cellular responses [84]. Regeneration is controlled by a different type of growth factors among which FGFs are the key players in tissue regeneration, including the neural, liver, bone, skin, intestine, cardiac, and muscle [85]. According to the amino acid sequence, the FGF family is divided into seven subfamilies [86]. However, FGF2 (basic FGF) is indicated to be widely applied for scarless wound healing and skin wound regenerative therapy [87]. It has been reported that the sustained release of basic FGF from a chitosan film as a delivery vehicle could accelerate wound healing in full-thickness skin wounds made on the backs of genetically diabetic mice and promote proliferation of fibroblasts and granulation tissue formation [52]. In another study, incorporation of bFGF with gelatin microspheres significantly accelerated dermal tissue regeneration [88]. Furthermore, studies have identified that FGFs are key regulators of keratinocyte migration in wounded skin, as the loss of FGFR1 and FGFR2 in keratinocytes results in a wound-healing defect [89].

### Hepatocyte growth factor (HGF)

The HGF was originally discovered as a mitogen of hepatocytes to be produced by stromal cells. HGF stimulates many properties of the epithelial cell, including proliferation, motility, morphogenesis, and angiogenesis via tyrosine phosphorylation of its receptor, tyrosine-protein kinase Met (c-Met) [90]. The mature form of HGF is composed of α/β heterodimers linked by a disulfide bond. The α-chain contains an N-terminal hairpin domain and first Kringle domain and exhibits a high-affinity binding site for Met, and the β-chain has a serine protease-like structure; although the α-chain is required for Met binding, it is not able to activate Met and the β-chain induces the activation of Met and biological responses [91]. The binding of HGF to its receptor, c-Met, results in structural changes in c-Met and phosphorylation of protein tyrosine kinase (PTK) domain. Subsequently, two other phosphotyrosines in the carboxy-terminal multifunctional docking domain recruit intracellular signaling molecules Grb2 (growth factor-receptor-bound protein 2), Gab1 (Grb2-associated binder 1), phosphoinositol 3-kinase (PI3K), MAPK, PLCγ (phospholipase Cγ) and Shp2 (SH2-domain-containing protein tyrosine phosphatase 2), signal transducer and activator of transcription factor (STAT) pathway [92, 93]. Therefore, c-Met and its related signaling pathways play a crucial role in the diverse process, including embryogenesis, wound healing, organ regeneration, and mature tissue survival [94]. It promotes mitogenic, morphogenic, and mitogenic activity in various cell types. HGF/Met contributes to immune regulation by modulation of DC migration and activation of monocytes and macrophages [95, 96]. HGF is a cytokine known to play multiple roles during the various stages of wound healing and accelerates wound healing by promoting the dedifferentiation of epidermal cells through 훽1-integrin/ILK pathway [97]. According to this study, HGF increased the expressions of the cell adhesion molecules 훽1-integrin and the cytoskeleton remodeling protein integrin-linked kinase (ILK) in epidermal cells in vivo and in vitro. Baek et al. [98] demonstrated that Met signaling in skin-resident DCs was essential for their emigration toward draining lymph nodes upon inflammation-induced activation. These findings were supported using a conditional Met-deficient mouse model where activated skin resident DCs failed to migrate toward the skin-draining lymph nodes despite an activated phenotype.

### Epidermal growth factor (EGF)

The EGF is primarily secreted by platelets, macrophages, fibroblasts, and keratinocyte and is present during dermal wound healing and facilitates skin regeneration. The binding of EGF to EGFR activates EFGR through ligand-induced dimerization, leading to a downstream signaling cascade, including Ras/MAPK, PLCγ/PKC, PI3K/Akt, and STAT [99, 100]. These signaling pathways are classified into four different categories: migration, proliferation, cytoprotection, and EMT among which migration and proliferation pathways have been required for wound healing [101]. EGF is influenced by different components of the keratinocyte migration machinery and induces contraction of keratinocytes, which are critical to wound re-epithelialization [102]. Despite extensive progress in the exogenous EGF in the treatment of acute wounds, its efficacy in chronic wound therapy is limited owing to their short half-life in vivo and poor transdermal permeability [103]. To overcome these restrictions, EGF was conjugated to an efficient delivery system to extend the residence time of EGF in the wound area and significantly regenerated skin tissue [104, 105].

## Development and application of stem cell technologies

The main clinical focus of stem cell application in wound care is to target improved quality of wound healing. The medical practitioner would anticipate achieving acceleration in healing, prevention of wound contracture and scar formation, earlier wound closure, and ideally regeneration of the skin and its appendages using stem cells [8]. Stem cells, defined based on the findings obtained by McCulloch and Till [7], are characterized by their capacity for self-renewal, asymmetric replication, and differentiation to other cells building different tissues and organs. Stem cells replenish lost cells throughout an organism’s lifespan. They have the capacity for unlimited replication providing a population of “sister” SCs. These cells are responsible for self-renewal and differentiate tissue-specific cells. This process maintains the constant number of aging somatic cells, which have become apoptotic. Their therapeutic potential is largely due to their capability to secrete pro-regenerative cytokines, causing them to be an attractive choice for the treatment of chronic wounds [6]. Among the main sources of cells that might be used for wound healing and regeneration of injured skin are embryonic stem cells (ESCs), induced pluripotent stem cells (iPS), and adult stem cells [106]. However, the remaining challenge of stem cell application for skin regeneration is still to describe the optimum source and the method of processing and administration from a clinical standpoint and to define the roles of stem cells in the real clinical situation [107]. Table 1 shows stem cells used for wound therapeutic.

**Table 1:** Stem cells used for in wound therapeutic

Stem cells types	Delivery mode	Wound types	Correction efficiency	Model source use	Treatment effect notes	Reference
BM-SCs	Scratch wound assay	Wound closure	3 days	Human	Stimulate fibroblasts, migration of keratinocyte and synthesis ECM proteins	[238]
BM-SCs	Tail vein injection post-operatively	Ischemia flap	7 days	Murine	Enhance angiogenesis and vascular regeneration	[141]
autologous MSC	Fibrin spray system	Cutaneous wound	12 weeks	Murine and human	Stimulate closure of full-thickness wounds in diabetic mice and wound healing repair	[239]
Combination hMSC with bFGF		Cutaneous wound	42 days	Rat	Increase re-epithelialization	[240]
Co-culture dermal fibroblasts with BM-SCs	Scratch wound assay	Wound closure	3 days	Murine	Increase proliferation and migration of dermal fibroblasts	[241]
MSCs	Subjection	Incision wound	4 days	Mice	Enhance tissue regeneration capacity especially in older populations	[242]
Autologous bone marrow	Aspiration	Chronic Wound	5 weeks	Human	Rebuilding of dermal	[138]
MSCs	Closed culture devices	Radiation burns	5 months	Human	Modulation radiation inflammatory process	[243]
Autologous MSCs	Injection	Diabetic ulcer	4 weeks	Human	Successful healing	[244]
Allogeneic BM-SCs	Intradermal	Excisional wound	14 days	Murine	Accelerate wound closure, increase re-epithelialization and angiogenesis	[245]
BM-SCs	Aspiration	Non-healing wound	5 days	Human	Increase synthesis of collagen	[246]
MSCs	Injection	Cutaneous wound	2 weeks	Human	Promote angiogenesis	[247]
MSCs	Mechanical loading	Incision wound		Mouse	Enhancement of angiogenesis	[248]
hUC-MSCs)	Transplantation	Burn	8 weeks	Rat	Decrease inflammatory cells, increase neovascularization and enhance collagen level	[249]
ASCs	Transplantation	Non-irradiated and irradiated	14 days	Mouse	Promote dermal wound healing, enhance wound closure and collagen secretion	[250]
hESCs	Grafting	Burn	Enhanced wound healing	Human-mice	hESC-derived epidermis showed a pluristratified structure, consistent with that of mature native human skin	[251]
ESCs	Directly on a gauze	Chronic Wounds	Accelerated wound healing	Diabetic mice	The beneficial effects were evident both histopathologically and immunohistochemically	[252]
Mouse-iPSCs	Grafting	Inherited skin disorders	Enhanced wound healing	Mouse	iPSC-KC stem cells were able to regenerate the epidermis, hair follicles, and sebaceous glands in an in vivo graft assay	[253]
hiPSC-MSCs-Exos	Injected locally	Injured tissues	Facilitated cutaneous wound healing	Human-rat	Accelerated re-epithelialization, reduced scar widths, the promotion of collagen maturity, promoted the generation of newly formed vessels, accelerated their maturation in wound sites	[254]
hiPSC	Grafting	Skin disease	Reconstitution of normal skin structures	Human-SCID mice	Skin appendages, such as hair follicles and glands, were not detected, and no cyst or tumor formation	[255]
hiPSCs	Grafting	Inherited skin disorder	Reconstitute human skin	EB patient-SCID mice	The reconstituted skin expressed human Col17 at the basement membrane zone, human type VII collagen and human keratin 14 were expressed in the basal layer	[256]

### Embryonic stem cells (ESCs)

The ESCs were first established from the inner cell mass (ICM) of mouse blastocysts in 1981, and the term “embryonic stem (ES) cell” was coined [108]. ES cells are pluripotent stem cells derived from the inner cell mass of the preimplantation blastocyst (35-day-old embryo) and obtained from mice, humans, and nonhuman primates. ES cells have the ability to differentiate cell types, including neural cells, blood cells, adipocytes, chondrocytes, muscle cells, and skin cells [109]. In an attempt to utilize the remarkable regenerative potential of ESCs for cutaneous repair, Guenou et al. showed that human embryonic stem cells growing in induction medium containing BMP4 (bone morphogenetic protein-4) and ascorbic acid could differentiate between basal keratinocytes, which were subsequently used to reconstitute the epidermis composed of multiple layers of differentiated cells. These tissues were also successfully transplanted into nude mice to facilitate wound healing [110]. In another report, Shroff et al. evaluated the effect of human embryonic stem cell (hESC) therapy in six patients with non-healing wounds. It showed that the wounds of all the patients healed after receiving hESC therapy. Reduction in the size of wounds and granulation was observed among all the patients [110]. Despite these promising findings, the use of embryonic stem cells has remained controversial. The cells could be the most suitable ones over adult stem cells for skin tissue regeneration owing to their capacity of self-renewal and the unlimited supply of differentiated keratinocytes or keratinocyte progenitors for treating cutaneous injuries [111]. In addition to the widespread clinical use of ESCs, which is currently elusive due to the potential for immunogenicity and tumorigenicity, another major limitation of using ESC-derived cells for regenerative wound healing is ethical controversy and substantial legal restrictions [7].

### Induced pluripotent stem cells (iPS cells)

The iPS cells are the newest class of pluripotent stem cells, which potentially combines the advantages of MSCs and ESCs, ushering in a new era of regenerative medicine [6]. In 2006, Yamanaka et al. [112] at Kyoto University in Japan observed that the introduction of four genes (Oct-3/4, Sox2, c- Myc, and KLF4) into cells from the mouse tail could reprogram the cell back to an embryonic state. In 2007, iPS cells were produced from human cells [113]. These induced pluripotent stem cells were shown to be remarkably similar to ESCs in morphology, proliferation potential, gene expression pattern, pluripotency, and telomerase activity. Like ESCs, iPSCs can differentiate between all types of cells from the skin to nerve and muscle [7]. This revolutionary technology allows for generation of autologous pluripotent stem cell populations, thereby circumventing the major limitations of ESC, including ethical concerns and potential for immunological rejection [113]. Taking advantage of these characteristics, significant progress has been made in the differentiation of iPSCs into skin cells—including folliculogenic human epithelial stem cells, fibroblasts, and keratinocytes—to engineer skin substitutes [106]. Bilousova et al. induced iPS cells in vitro to differentiate skin-like cell lines and to form multi-differentiated epidermis, hair follicles, and sebaceous glands [114]. Additionally, Itoh et al. [115] generated in vitro 3-D skin equivalents exclusively composed of human iPSC-derived keratinocytes and fibroblasts. Two recent studies conducted by Umegaki-Arao et al. [116] and Sebastiano et al. have further proven this concept. One of the most recent studies in this regard suggested that exosomes derived from human-induced pluripotent stem cell-derived mesenchymal stem cells (hiPSC-MSCs) facilitated cutaneous wound healing in rats by promoting collagen synthesis and angiogenesis [117]. However, despite experimental evidence supporting the therapeutic benefits of iPSCs, there are still numerous issues such as associated cancer risk development through using retroviral vectors, epigenetic memory retained from parent cells, genetic instability, inefficient cell re-programming yielding low cell numbers with high processing costs, and potential immunogenicity [118]. Therefore, iPSC-based therapies for wound-healing applications require further extensive analyses for safety and reliability of the reprogramming technology [119].

### Adult stem cell

The most stem cells used in skin regeneration and wound healing are adult stem cells owing to containing significant proliferative capacity, long-term self-renewal potential, and having the ability to differentiate into other lineages. They are found in various tissues, including the skin, heart, liver, brain, and bone marrow. Among the different types of adult stem cell, mesenchymal stem cells (MSCs) and adipose-derived stromal cells (ASCs) have gained considerable attention as a suitable candidate to enhance tissue regeneration [120].

#### Mesenchymal stem cells (MSCs)

The MSCs harvested from various sites (bone marrow, adipose tissue, amniotic fluid, and dermis) are considered a source for therapeutic approaches owing to their multilineage differentiation, high frequency, facility of isolation and characterization, and the ability of MSCs to migrate to injury sites in the body [121]. These cells are involved in all three phases during the wound-healing process. They also enhance wound healing by immune modulation, production of growth factors, which enhance neovascularization and re-epithelialization, stimulate angiogenesis, and accelerate wound closure [122]. One case study has reported that increased wound closure occurs when MSCs are administrated and accelerated dermal fibroblast and keratinocyte migration [123]. Furthermore, Nakagawa et al. [124] suggested that the combination of hMSCs with bFGF in a skin defect model improved cutaneous wound healing as the hMSCs transdifferentiate into the epithelium. Smith et al. [125] showed that MSCs secreted soluble factors inducing dermal fibroblast proliferation, migration, and chemotaxis. Endogenous bone marrow-derived mesenchymal stem cells in the dermis may provide an important early signal for dermal fibroblast responses to cutaneous injury. Li et al. [126] demonstrated that activated MSCs promoted wound healing in acute incisional wounds, as reflected in regained tensile strength.

A clinical study was performed to test a new technique for the treatment of chronic non-healing wound (diabetic ulcer) using autologous graft composed of autologous skin fibroblasts on biodegradable collagen membrane (Coladerm) in combination with autologous MSC derived from the patient’s bone marrow. The wound showed a steady overall decrease in wound size and an increase in the vascularity of the dermis and in the dermal thickness of the wound bed after 29 days of combined treatment [127]. The treatment of burn injuries, especially severe ones, has always been a challenging issue, but the use of MSCs had beneficial therapeutic effects on burns wound healing. A case report of radiation burns has indicated the efficiency of a new therapeutic approach combining surgery and local cellular therapy using autologous MSCs, which this benefit of the local cell therapy could be linked to the “drug cell” activity of MSC by modulating radiation inflammatory processes [128]. During the normal wound-healing process, angiogenesis is one of the most important stages in which MSCs secret various pro-angiogenic factors such as VEGF to promote endothelial cell proliferation and form new vessels [129]. There is evidence that suggests topical VEGF accelerates diabetic wound healing through increased angiogenesis as well as mobilizing and recruiting bone marrow-derived cells [73]. Han et al. [130] compared proliferation, collagen synthesis, and growth factor production of human BSCs as important contributing factors for wound healing, to those of dermal fibroblasts in vitro. There were no significant differences in cell proliferation and TGF-β production. However, BSCs produced much higher amounts of collagen, bFGF, and VEGF. Recently, a study has observed that local transplantation of MSCs improves cutaneous wound healing via VEGF-paracrine secreted from MSCs [131]. Kasper et al. [132] revealed that mechanical loading of MSCs seemed to result in a paracrine stimulation of angiogenesis, most likely by regulating a network of several angiogenic molecules. Experimental studies established that MSCs could orchestrate the inflammatory response following tissue injury. Transplantation of human umbilical cord MSCs into cutaneous rat wounds significantly accelerated wound healing and remarkably decreased the quantity of infiltrated inflammatory cells and levels of IL-1, IL-6, and TNF-a and increased levels of IL-10 and TSG-6 in wounds. Additionally, hUC-MSCs increased the level of VEGF in severe burn wounds and promoted wound angiogenesis [133]. Aggarwal and Pittenger in their study described that MSCs were capable of modulating allogeneic immune cell responses through reducing the secretion of TNF-α and interferon-γ (IFN-γ) [134]. Undoubtedly, many studies have outlined that mesenchymal cells are considered suitable candidates for cell-based therapeutic approaches, but in spite of developments in MSC-based therapy, there are a number of limitations in the utilization of MSCs. One potential limitation in the application of MSCs for treatment is their poor viability following implantation, curtailing a long-term safety profile. However, some strategies have been developed to improve the survival of the transplanted MSCs [135]. The self-renewal capability of MSCs and their molecular mechanism are unknown, and it is still unclear to identify how culture expansion alters the cellular composition and function of populations [136].

##### Bone marrow-derived stem cells (BM-SCs)

BM-SCs are considered the primary source of MSCs in adults and a good candidate for the treatment of different types of wounds [137]. Preclinical studies using autologous BM-MSC have reported the potential therapeutic effect of these cells in dermal rebuilding and scarring reduction in chronic wound [138]. A study of patients with non-healing ulcers of the lower limb found that application of BM-MSC led to significant improvement in pain-free walking distance and reduction in ulcer size [139]. BM-MSCs have been confirmed to improve indicators related to wound healing through increasing re-epithelialization and thickness of the regenerated epidermis [140]. Wan et al. [141] demonstrated that transplantation of allogeneic bone marrow-derived mesenchymal stem cells could promote the delayed wound healing in diabetic rats. Falanga et al. [142] stated that the cultured autologous BM-MSC delivered to wounds using a fibrin spray system could achieve healing in murine and human cutaneous wounds. A study on 8 patients, whose non-healing diabetic ulcers were treated with a combination of bone marrow stem cells, platelets, fibrin glue, and collagen matrix, presented successful healing for three patients and a significant reduction in the remaining five patients [143]. Wu et al. discovered that BM-MSCs enhanced wound healing in nondiabetic and diabetic mice by promoting re-epithelialization, cell infiltration, and angiogenesis. Moreover, a study proved that circulating bone marrow-derived MSCs home to perivascular sites in critically ischemic tissue exhibited paracrine function and augmented microhemodynamics. These effects were mediated through arteriogenesis and angiogenesis, which contributed to vascular regeneration [144]. Although BM-MSC is successfully implemented in clinical treatment, other limitations in therapeutic efficacy are challenges that need to be addressed through an extensive investigation of BM-MSC. The risks of BM-MSC during clinical translation are harvesting invasiveness, in vitro culture, and further cost-time resource.

##### Human umbilical cord-derived mesenchymal stem cells (UC-MSCs)

UC-MSCs show promising therapeutic effects due to immunological compatibility, long-term survival, multi-directional differentiation potential, and easy isolation [145]. In vitro experiments have demonstrated that treatment of diabetic wounds with hUCB-MSCs shows higher cell proliferation and collagen synthesis compared to fibroblasts [146]. A similar observation reported that transplantation of UC-MSC accelerated wound closure in diabetic mice. Although many clinical trials have not been developed on using UC-MSCs in wound healing, they have advantages over BM-MSCs, including easy preparation, high number of cells from the cord, production large yield of MSCs, and retardation of senescence [147]. Despite the considerable therapeutic potential for stem cell to treat various diseases, there are still concerns about potentially dangerous consequences. One challenging area concerns the patient’s immune system. The challenges are also unusual since they mostly pertain to embryonic stem cells, whereas adult stem cells can alleviate immunological challenges that tend to accompany embryonic stem cells. Stem cells have the potential to divide many times and differentiate many cell types, which is their considerable promise. Paradoxically, owing to these abilities, stem cells also have the potential to form tumors. The possibility of transplanted stem cells differentiating into the wrong type of tissue is yet another concern regarding therapeutic stem cell use. Stem cell lines used for research are not always “pure”, because their exposure to other animal cells to maintain viability causes contamination.

### Adipose-derived stem cells (ASCs)

ASCs are pluripotent cells with the ability to differentiate between various cell types. These cells have advantages over MSCs, including their high accessibility with minimal invasiveness and no ethical limitations [148]. ASCs can promote wound healing and trigger neovascularization through their ability to differentiate endothelial cells and release VEGF [149]. Another study presented that hypoxia increased the proliferation of ASCs and enhanced the wound-healing function of ASCs, at least partly, by upregulating the secretion of VEGF and bFGF [150]. Kim et al. [151] investigated that ASCs had effects on human dermal fibroblasts (HDFs) by increasing collagen synthesis and promoting proliferation of HDFs, suggesting that ADSCs could be used for the treatment of wound healing. Furthermore, it has been illustrated that AD-MSCs possess considerable anti-inflammatory and angiogenic potential, in which due to these properties, they can be distinguished from dermal fibroblasts [152]. Such advancements demonstrate that ADSCs are extremely promising as an alternative tool for the regenerative strategy for wound therapy.

## MSCs and immune modulation

For the first time in 2002, Bartholomew et al. indicated that MSCs had the ability to modulate immunosuppression. They also showed prevention of rejection in a baboon skin allograft model in vivo and suppression of a mixed lymphocyte response in vitro [153]. Considering the fact that the MSCs immune response properties were reported for the first time, subsequent studies have demonstrated that MSCs mediate immunosuppression in human and animal models.

Regarding the successful preliminary clinical outcomes, the mechanisms concerned with MSC interactions with the immune response as understood today are worth mentioning. MSCs can interact with various immune cells such as T cells, B cells, natural killer (NK) cells, DCs, neutrophil, and macrophages [154]. The interaction mechanisms were indicated to depend on cell-cell contact working in cooperation with the secretion of soluble immune factors in order to induce MSC-regulated immunosuppression [155]. Particular modulators, such as multitude of immune-modulatory factors, growth factors, and cytokines, balance immune profiles and modulate inflammatory responses [156]. In other words, soluble immune secretomes such as 3-dioxygenase (IDO), prostaglandin E2 (PGE-2), nitric oxide (NO), and indoleamine 2 respond to immune cells in order to activate immunoregulation through MSCs [157].

Intracellular secretomes, the main histocompatibility complex (MHC) antigens, and adhesion molecules are all necessary to induce immune suppression. In this regard, the Fas ligand/Fas receptor interaction (FasL/FasR) and T cells play a crucial role in the function of T cell reaction [158]. Extracellular vesicles produced by MSCs facilitate generating regulatory T cells and M2 macrophages while suppressing proliferation of B cells and T cells and maturation of monocytes [159].

In addition, MSCs are able to repair damaged cells and tissues and regulate inflammatory progress by adhering to inflammatory sites [160]. Integration of MSC with inflammatory actions can restrain and fortify the immune response and relies on the kinds of inflammatory secretomes, the function of immune suppressants, and the immune system general condition [161]. As an interesting point, when MSCs are stimulated by inflammatory cytokines such as interleukin- (IL-1) and tumor necrosis factor (TNF), they only modulate immunosuppression [162]. MSCs not only produce immune-regulatory secretors mediating the inflammation process but also respond to inflammatory cytokines. For instance, many chemokines produced by MSCs, indoleamine 2,3-dioxygenase (IDO) in humans, and nitric oxide (NO) in mice play a major part in MSC-mediated immunomodulation [163]. Additionally, MSC secretomes such as tumor-specific glycoprotein (TSG6) and growth factors HGF have been effectively used for the treatment of immune diseases [164]. In this respect, MSCs have also been employed to effectively treat patients with severe immune disorders such as Crohn’s disease and SLE [165].

### Immune cells interact with MSCs in immunomodulation

**Fig. 1:**
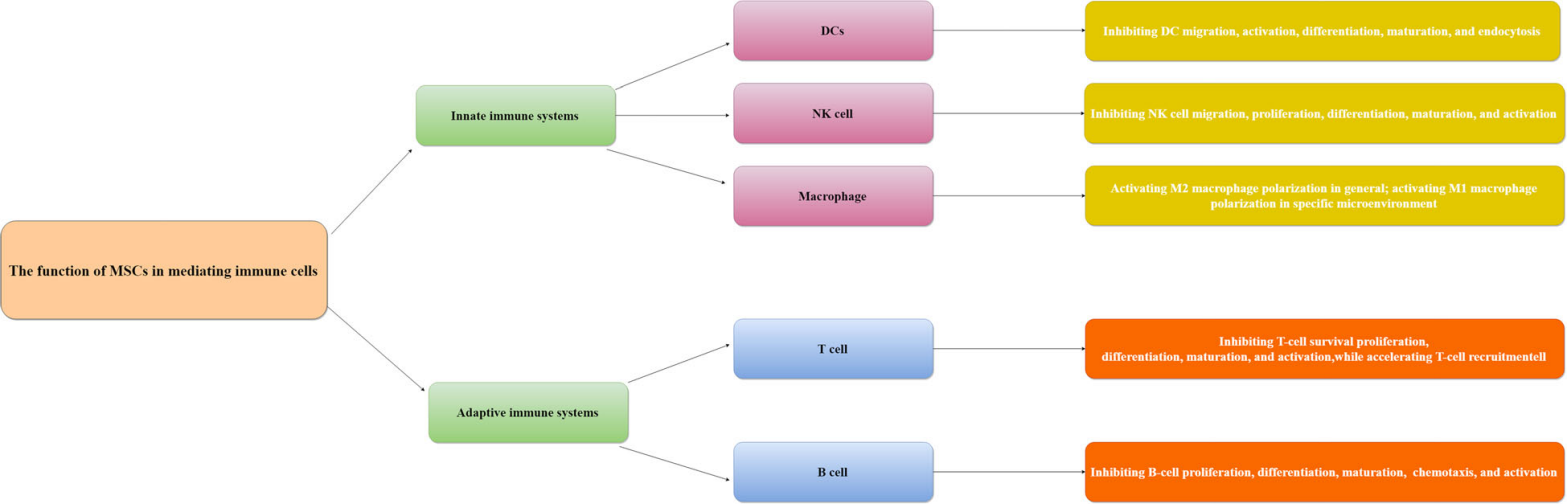
The function of MSCs used in innate and adaptive immune systems

Both in vitro and in vivo studies have indicated that MSCs demonstrate their multipotency as an immunomodulation mediator. MSCs significantly affect immunosuppression through refraining immune cells in both adaptive and innate immune systems (Fig. 1).

#### Innate immunity

The innate immune system plays a crucial role not only in the elimination of pathogens targeted by an adaptive immune response but also in the adaptive immune reaction [166]. NK cells, DCs, and macrophages form the innate immune system, and their interaction with MSCs inhibits inflammatory responses and improves regenerative processes [167].

##### (A) Myeloid dendritic cells (DCs)

DCs modulate and maintain immune responses through the activation of cells in the innate immune reaction following DC maturation and acceleration of antigen-specific T cell processes [168, 169]. Recent studies have indicated that MSCs have immunosuppressive functions on DCs through decreasing the cell-surface expression of CD1-α, CD40, CD80, CD83, CD86, and MHCII, and restraining DC differentiation from monocytes [170]. DCs, after incubation with MSCs, would lose their ability to motivate lymphocytes by accelerating IL-10 release and downregulating interferon-γ (IFN-γ) as well as TNF-α expression [171]. In this respect, the Notch pathway relying on IFN-γ-secretase mediates the MSC-DC interaction [172]. PGE-2 seems to regulate the molecular mechanisms of MSCs restraining DC maturation [173]. Furthermore, MSCs are able to damage DCs migration through presenting antigens for activating T cells and suppressing molecules tied to DCs [174, 175]. By inhibiting TNF formation, MSCs can depress the DCs proinflammatory capacity [176]. The important point is that MSC inhibitory effects play a crucial role in relieving a number of immune disorders, including allograft rejection [172], type 1 diabetes, and acute GVHD [177, 178].

##### (B) Natural killer (NK) cells

Natural killer (NK) cells have cytolytic activity and produce proinflammatory cytokines [179]. By immunosuppressive secretors such as PGE2, TGF-β, and sHLA-G, MSCs inhibit the effects of NK cells, leading to reduction of IFN-γ secretion and induction of cytotoxic effects against virus-infected cells [180]. Suppressing the activating NK cell receptor expression completes this inhibitory action, which is mediated by PGE-2 and IDO [181]. In addition to these findings, direct cell-cell contact plays a particular role in suppressing NK cells being associated with expression of Toll-like receptor- (TLR-)4 on MSCs [182]. By suppressing the secretion of NKp30 and NKG2D, as the surface receptors associated with NK cell activation, MSCs improve cytotoxic movement [183]. Nevertheless, the potent suppressive actions of MSCs appeared only at high MSC-to-NK ratios [180]. Moreover, it has been indicated that activated NK cells are able to dissolve MSCs in the case existence of activating receptors on NK cells [184]. Overall, these discoveries demonstrate that interaction between NK cells and MSCs is dependent on the ratios of both cells and their microenvironment [185].

##### (C) Macrophages

It is clearly demonstrated that macrophages are significant cells in the innate immune system with high plasticity [186]. In this regard, macrophages, according to the specific microenvironment of MSCs, may be polarized into classically activated M1 macrophages or alternatively activated M2 macrophages [187]. In general, by releasing various chemokines and inflammatory cytokines, M1 macrophages possess prominent antimicrobial properties, whereas M2 macrophages are capable of alleviating inflammation and expediting tissue repair through secretion of IL-10 and trophic factors [188]. Furthermore, the coculture of macrophages with MSCs induces production of M2 macrophages, downregulating levels of inflammatory cytokines, such as IFNγ, TNF-α, IL-1β, and IL-12, as well as upregulating the phagocytic activity and secretion of IL-10 [189, 190]. Recent studies have reported that by responding to TLR4 ligation, then inducing monocyte emigration, MSCs accelerate monocyte chemotactic protein-1 (MCP1) secretion [191]. In a zymosan-induced peritonitis injury model, by secreting TNF-stimulated gene 6 (TSG6), human MSCs activate peritoneal macrophages, regulating TLR2 nuclear factor-κB (NF-κB) signaling [192]. Furthermore, MSCs have been demonstrated to improve immune disorders and facilitate tissue regeneration through increasing the macrophages concentration at injury locations [193, 194].

#### Adaptive immunity

The adaptive immune system has its own specific properties, particularly immunological memory and antigen-specific immune response. The system is composed of CD4+ T helper and CD8+ cytotoxic T lymphocytes transmitting an appropriate antigen-specific immune response after antigen-presenting cells (APCs) undergoing antigen processing and presentation [166].

##### (A) T cells

T cells are mostly distributed in both human and animal tissues and, once activated, can differentiate between T helper (Th) 1, regulatory T cell (Treg) subpopulation, Th2, Th9, or Th17, according to the cytokine microenvironment and the stimulation intensity [195, 196]. It has been indicated that MSCs tightly interact with T cells [197]. In this regard, T cells, as a key mediator of the adaptive immune system, protect organisms from infections and malignancies, as well as modulate different autoimmune diseases [197].

Furthermore, MSCs secrete a considerable quantity of chemokines, immunosuppressive factors, and adhesion molecules, being responsible for effective T cell suppression, involved in T cell apoptosis, differentiation, and proliferation [198]. For instance, MSCs are able to repress T cell proliferation via cellular or nonspecific mitogenic stimuli [199] and promote activated T cell apoptosis through the Fas/Fas ligand pathway [200]. MSCs constitutively secrete coinhibitory molecule HLA-G and B7-H4, presenting an immunosuppressive action on T cell and influencing their T cell-mediated cytotoxicity and proliferation [201]. Nevertheless, MSCs immunosuppressive capacity is not activated at all times and is dependent on the type and strength of the inflammatory stimulation [202]. MSCs do not restrain T cell proliferation in the presence of pathogen-associated molecules and TLRs such as TLR4 and TLR3 damaging Notch signaling, as a consequence, recovering effective T cell to respond to pathogens [203]. Furthermore, as a specialized subset of T cells, regulatory T cells restrain the effects of the immune system, resulting in sustaining homeostasis and relieving their own antigens [204].

##### (B) B cells

B cells are considered the second major cell genre associated with adaptive immune responses. The cells resist and hunt down outside pathogens through producing specific antibodies [205, 206]. Both human and murine MSCs are able to inhibit B cell activation and proliferation in vitro [207]. Furthermore, MSCs also suppress expression of chemokine receptors and differentiation of B cells due to secretion of soluble molecules and cell-cell contact [208]. Metalloproteinase-processed CC-chemokine ligand 2(CCL2) released by MSCs suppress activator of transcription 3 (STAT3) activity and signal transducer, leading to downregulating Paired box 5 (PAX5), thereby inhibiting immunoglobulin synthesis [209]. A number of other signaling pathways such as extracellular response kinase ½, p38, Akt signaling, and B lymphocyte-induced maturation protein 1 (Blimp1) modulate B cell activation [210]. Nevertheless, insufficient inflammatory signal-activated MSCs in patients with SLE may support differentiation and proliferation of antibody-releasing B cells [211]. Overall, MSCs suppress antibody production by B cells; this effect depends on the MSCs to B cells ratio and the inflammatory stimulation strength [212, 213].

### Soluble factors secreted by MSCs in immunomodulation

**Fig. 2:**
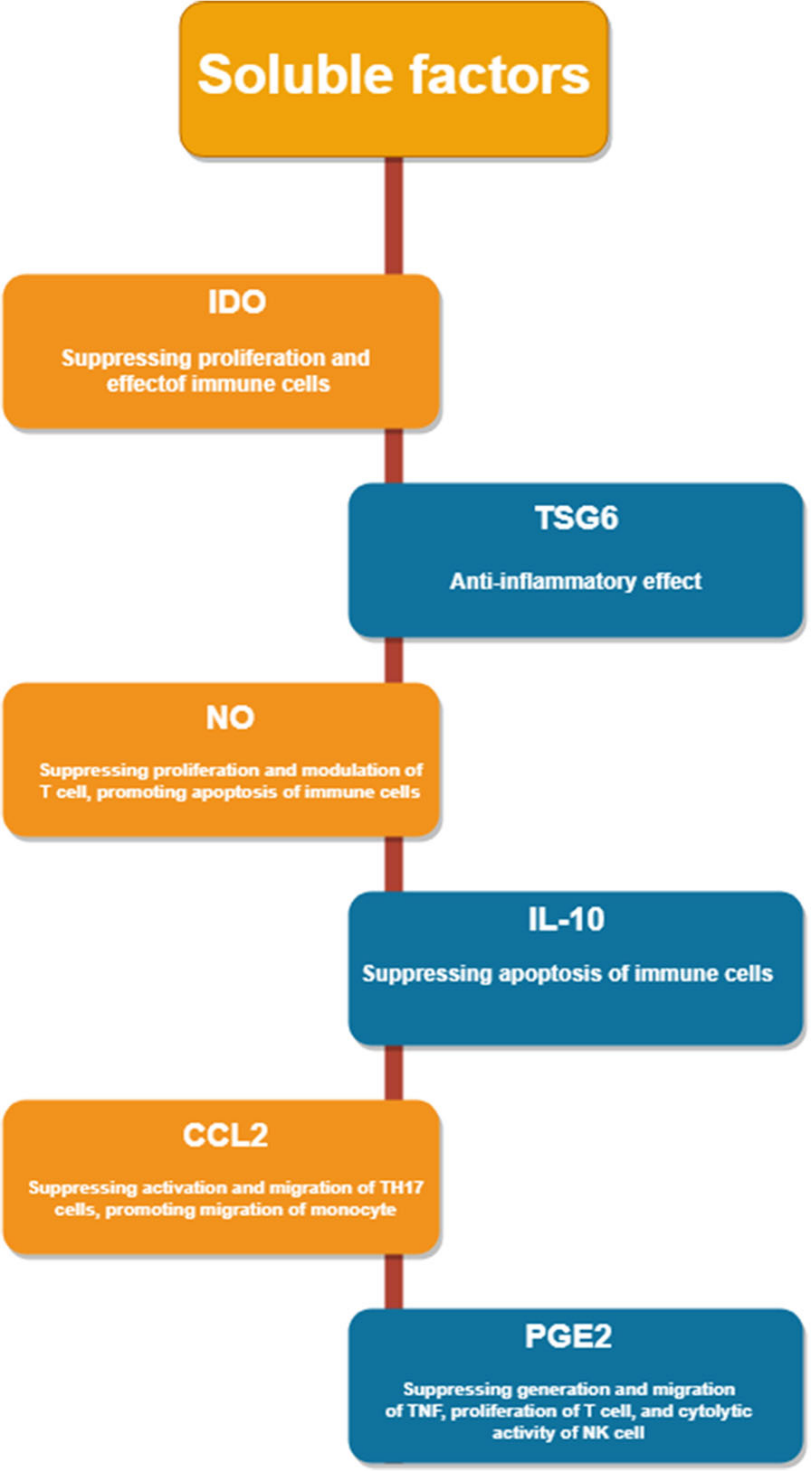
Biological function of MSCs in soluble factor secreted

By secreting multiple soluble immune factors, MSCs could interact with immune cells in both innate and adaptive immune systems to induce MSC-regulated immunosuppression [214]. During an immune response, some soluble factors are released by MSCs, such as growth factors, cytokines, chemokines, and hormones, which act on immune cells and exert their functions through suppressing immunology activity and repairing damaged cells [215, 216] (Fig. 2). Owing to an inflammatory cytokine-licensing process by MSCs, the inflammatory response is essential for MSCs to exert effects on immunomodulation. As a result, MSC immunoregulatory activities require inflammatory cytokines secreted by T cells and antigen-presenting cells, including IL-1α, interferon- (IFN-) γ, TNF-α, and IL-1β [217]. These inflammatory cytokines can activate MSCs to secrete immunosuppressive factors composed of TSG6, IDO, IL-10, NO, galectins, CCL2, TGF-β, and PGE2 and then modulating tissue homeostasis [159, 218].

#### Indoleamine 2,3-dioxygenase (IDO)

It has recently been reported that by suppressing various immune cells such as T cells and NK cells, IDO mediates immunomodulation [212, 219]. IDO can restrain the effect and proliferation of immune cells through transforming tryptophan into its metabolite kynurenine [220]. In addition, IDO secreted by MSCs is capable of suppressing allogeneic T cell reactivity and promoting kidney allograft tolerance [221]. Furthermore, IDO has been suggested to be one of the immunosuppressive molecules representative for human MSCs [212, 222].

#### TNF-stimulated gene 6 (TSG6)

TSG6 has anti-inflammatory effects as a multifunctional protein [223]. Proinflammatory mediators like IL-1 and TNF-a may stimulate TSG6 secretion [224]. It has been reported that in a mouse model of myocardial infarction, microembolization induces TSG6 to interact with damaged lung. Therefore, TSG6 plays a crucial role in enhancing cardiac function, as well as reducing inflammation and infarct size [225].

#### NO

MSCs, in the presence of proinflammatory cytokines, accelerate high expression of inducible NO synthase (iNOS), stimulating the secretion of NO, and leading to inhibition of T cell proliferation [226]. Both in vitro and in vivo studies indicated that murine MSCs lacking iNOS showed reduced inhibition capability [160]. Interestingly, NO high concentrations may suppress immune modulation and result in immune cell apoptosis through inhibiting signal transducer in T cells and signal transducer and activator of transcription 5 (STAT5) phosphorylation [226, 227]. Nevertheless, NO is an extremely unstable oxidative molecule, and both chemokines and adhesion molecules can contribute to it in exerting immunosuppressive action [211, 228].

#### IL-10

IL-10 has been reported to play a significant part in MSC-regulated immunosuppression [134]. Antigen-presenting cells such as dendritic cells and monocytes could work with MSCs to induce IL-10 secretion [229]. Furthermore, by stimulating E prostanoid receptors, macrophages can deliver large quantities of IL-10, thereby protecting tissues against neutrophils migration [230].

#### CC-chemokine ligand 2 (CCL2)

As a metalloproteinase-processed chemokine, CC-chemokine ligand 2 (CCL2) antagonizes the function of CC-chemokine receptor 2 (CCR2), being the cognate receptor of CCL2 [231]. Binding of CCL2 to CCR2 has been demonstrated to mediate immunosuppression of MSCs through inhibiting migration and activation effects on TH17 cells in experimental autoimmune encephalomyelitis (EAE) [232]. Moreover, CCL2 secreted by mouse MSCs facilitate monocyte migration from the bone marrow into the blood stream, confirming the point that interaction of MSC with innate immune responses influences the immune system [232].

#### Prostaglandin E2 (PGE2)

Another immunosuppressive factor secreted by inflammatory stimulus-induced MSCs is PGE2, regulating immunosuppression of MSCs in macrophages, DCs, T cells, and NK cells [217, 219]. In vitro, PGE2 produced by mouse MSCs restrain some cell functions like TNF migration and generation [233]. In addition, IL-10-dependent PGE2, in an experimental mouse model of sepsis, has been indicated to play a crucial role in effectively treating mice with MSCs [217]. More importantly, PGE2 cooperates with IDO and exerts immunosuppressive actions in human MSCs like inhibiting NK cell cytolytic activity and T cell proliferation [219]. It seems that depending on the inflammatory microenvironment, all these molecules exert their functions. Thus, future research should focus on mediator mechanisms regulating the immunosuppressive characteristics of MSCs and their local microenvironments, providing a wide perspective for therapeutic application of MSCs [218].

## An outlook on the future

The aim of skin regeneration is to achieve structural and functional reconstruction, reduce scar formation, and improve the quality of wound healing. Stem cell-based therapy has offered a novel and powerful strategy in burns and wound management. Stem cells have been demonstrated to have considerable potential in skin tissue regeneration, as these cells can not only regenerate lost tissue but also promote wound repair through a paracrine manner. Several cell types such as embryonic stem cells, iPSCs, and mesenchymal stem cells are currently under intense investigation [111]. The availability of adult stem cells and iPS cells in the patient provides opportunities for generating these structures without the risk of immune rejection [7]. Although advances in the field of hiPSCs have grown exponentially, much still needs to be understood and improved upon in terms of the reprogramming process itself, the differentiation potential of cells, the difference between iPSCs and ESCs, “dark side” to induced pluripotency, and their future use in clinical therapy [234, 235]. Recent data on the MSC therapy in cutaneous repair have showed several reasons why mesenchymal stem cells provide unique and effective support for stimulating the wound-healing process in a chronic wound. Ultimately, these cells have the ability to suppress excessive inflammation and reduce scarring while stimulating de novo angiogenesis in the wound bed, all leading to promising outcomes in chronic wound repair [236]. Despite the rapid progress in evaluating the efficacy of MSC transplantation for wound healing, several questions still need to be addressed. Further studies are necessary to characterize the niche of MSC, which helps MSCs to be effective in the wound-healing process. Further investigation of the experimental and clinical application of stem cells in wound healing is necessary to identify the ideal source of stem cells and the most efficacious mode of cell delivery [111]. The use of stem cells has been partially effective; however, the potential risks of malignant teratoma formation and long-term adverse effects of the stem cells should be considered, and more extensive studies are required in this regard [237]. In addition, there is a lack of information on long-term outcomes of skin wound treatment using such regenerative therapies. Nevertheless, for all the aforementioned reasons, researchers should be encouraged to increase the knowledge of cell-based regenerative therapies, and future studies should focus on developing a solid therapy for the treatment of skin wounds in mammals [59]. We maintain that these problems will certainly be resolved by developments in cell biology, tissue engineering, and regenerative medicine.

## Conclusion

Wound healing has always been the most challenging issue owing to the presence of various cell and molecules working in an orchestrating way. Any disorder can cause healing failure and result in progression of an acute wound to a chronic wound. Thus far, various procedures have been employed in the treatment of skin ulcers among which cell-based therapy particularly adult stem cell has emerged as a promising treatment to promote scarless wound healing*.* Through the capability of mesenchymal stem cells in immunomodulation and tissue regeneration, they have received particular attention to other adult stem cells. Clinical data demonstrated that autologous MSC transplantation promoted healing in all wound repair phases. However, harvesting and isolating an optimized pool of MSC with high purity obstructs the progress of developing new therapies. Thus, the characterization of MSCs with niche-specific factors still remains a challenge for researchers. To overcome these limitations, understanding of cellular and molecular mechanisms underlying stem cell action is necessary. Subsequently, improvement methods of stem cell delivery and identification of the ideal source are needed for clinical application of these cells in wound healing.
